# Supplementary material for: Capturing individual variation in children’s electroencephalograms during nREM sleep
Source: PLoS Comput Biol. 2026 Jan 30;22(1):e1013931. doi: 10.1371/journal.pcbi.1013931 (PMC12885382; doi:10.1371/journal.pcbi.1013931)
Supplement: S1 Table — Estimates include robust standard errors with 95% confidence intervals. (PDF) [file pcbi.1013931.s001.pdf]

Table S1. Polynomial regression of N1 area under the curve (AUC) of PSD and age.

	Coef.	SE	t	$P > \|t\|$	CI [0.025	0.975]
Intercept	-99.88	8.48	-11.77	<0.001	-116.54	-83.23
Age	52.23	6.25	8.36	<0.001	39.97	64.49
Age^2	-9.92	1.03	-9.60	<0.001	-11.95	-7.89
Age^3	0.38	0.05	8.38	<0.001	0.29	0.47
**Model Statistics**						
R-squared			0.354			
Adj. R-squared			0.351			
F-statistic			157.5	$p < 0.001$		
No. Observations			760			

Estimates include robust standard errors with 95% confidence intervals.
