# Supplementary material for: Capturing individual variation in children’s electroencephalograms during nREM sleep
Source: PLoS Comput Biol. 2026 Jan 30;22(1):e1013931. doi: 10.1371/journal.pcbi.1013931 (PMC12885382; doi:10.1371/journal.pcbi.1013931)
Supplement: S2 Table — Estimates include robust standard errors with 95% confidence intervals. (PDF) [file pcbi.1013931.s002.pdf]

Table S2. Polynomial regression of N2 area under the curve (AUC) of PSD and age.

	Coef.	SE	t	$P > \|t\|$	CI[0.025	0.975]
Intercept	-117.13	8.02	-14.61	<0.001	-132.87	-101.39
Age	60.97	5.91	10.33	<0.001	49.38	72.57
Age^2	-11.02	0.98	-11.78	<0.001	-12.94	-9.10
Age^3	0.43	0.04	10.09	<0.001	0.35	0.52
**Model Statistics**						
R-squared			0.314			
Adj. R-squared			0.311			
F-statistic			107.6	$p < 0.001$		
No. Observations			760			

Estimates include robust standard errors with 95% confidence intervals.
