# Supplementary material for: Capturing individual variation in children’s electroencephalograms during nREM sleep
Source: PLoS Comput Biol. 2026 Jan 30;22(1):e1013931. doi: 10.1371/journal.pcbi.1013931 (PMC12885382; doi:10.1371/journal.pcbi.1013931)
Supplement: S3 Table — Models are tabulated for both within and mixed sleep stage models. Fingerprinting task refers to the data used for training and validating the BRRR model with 10% of the population used as validation set. Success rate (SR) and proportion of total variance explained (PTVE) were averaged over the folds. The best results are highlighted. (PDF) [file pcbi.1013931.s003.pdf]

Table S3. The 10-fold CV results of different BRRR models.

Group	Fingerprinting task	All		>7 year olds	
		SR	PTVE	SR	PTVE
Within sleep stage	$\mathbf{N1_a+N1_b}$	0.73	0.77	**0.89**	**0.78**
	$N2_a+N2_b$	0.88	0.85	0.87	0.82
	$\mathbf{N2_a+N2_b+N2_c}$	**0.90**	**0.80**	0.89	0.75
Mixed sleep stage	$N1_a+N2_b$	0.24	0.60	0.44	0.57
	$\mathbf{N1_a+N1_b+N2_a}$	**0.72**	**0.64**	**0.90**	**0.61**
	$N1_a+N2_a+N2_b$	0.29	0.60	0.54	0.56

Models are tabulated for both within and mixed sleep stage models. Fingerprinting task refers to the data used for training and validating the BRRR model with 10% of the population used as validation set. Success rate (SR) and proportion of total variance explained (PTVE) were averaged over the folds. The best results are highlighted.
