# Supplementary material for: Capturing individual variation in children’s electroencephalograms during nREM sleep
Source: PLoS Comput Biol. 2026 Jan 30;22(1):e1013931. doi: 10.1371/journal.pcbi.1013931 (PMC12885382; doi:10.1371/journal.pcbi.1013931)
Supplement: S4 Table — Results are organized by descending success rate of BRRR fingerprinting. Corr refers to correlational fingerprinting. Bolded entries are those that are illustrated in result figures. (PDF) [file pcbi.1013931.s004.pdf]

Table S4. Fingerprinting results on whole data.

Fingerprinting task	PTVE$_{BRRR}$	SR$_{BRRR}$	SR$_{Corr}$
$N2_b$+$N2_c$	0.85	0.8	0.70
**$N2_a$+$N2_b$**	**0.85**	**0.8**	**0.69**
$N2_c$+$N2_d$	0.83	0.74	0.63
**$N1_a$+$N1_b$**	**0.78**	**0.56**	**0.44**
**$N2_a$+$N2_d$**	**0.73**	**0.36**	**0.22**
$N1_a$+$N2_a$	0.62	0.16	0.09
**$N1_a$+$N2_b$**	**0.6**	**0.10**	**0.05**
$N1_a$+$N2_d$	0.57	0.07	0.06

Results are organized by descending success rate of BRRR fingerprinting. *Corr* refers to correlational fingerprinting. Bolded entries are those that are illustrated in result figures.
