# Supplementary material for: Capturing individual variation in children’s electroencephalograms during nREM sleep
Source: PLoS Comput Biol. 2026 Jan 30;22(1):e1013931. doi: 10.1371/journal.pcbi.1013931 (PMC12885382; doi:10.1371/journal.pcbi.1013931)
Supplement: S5 Table — Results are ordered by descending success rates on all data. Results are shown for models trained with all data, with success rates separately for over 7-year old sub-population. Success rates are shown also for models trained with only >7 year olds. Latent space refers to data segments used in learning the latent representation, whereas fingerprinting task refers to segments projected to latent space that fingerprinting is then performed on. (PDF) [file pcbi.1013931.s005.pdf]

Table S5. Summary of the generalizability of different BRRR models.

		All data		>7 y.o
**Latent space**	**Fingerprinting task**	**SR**(All)	**SR**(>7 y.o.)	**SR**
$N2_a+N2_d$	$N2_b+N2_c$	0.80	0.89	0.76
$N1_a+N2_d$	$N2_a+N2_b$	0.74	0.82	0.72
$N2_a+N2_b$	$N2_c+N2_d$	0.74	0.82	0.69
$N1_a+N1_b$	$N2_a+N2_b$	0.71	0.80	0.69
$N2_a+N2_d$	$N1_a+N1_b$	0.54	0.63	0.76
$N2_a+N2_b$	$N1_a+N1_b$	0.48	0.58	0.72
$N1_a+N1_b$	$N2_a+N2_d$	0.19	0.29	0.25
$N1_a+N2_b$	$N1_b+N2_a$	0.27	0.42	0.41
$N2_c+N2_d$	$N1_a+N2_a$	0.11	0.15	0.25

Results are ordered by descending success rates on all data. Results are shown for models trained with all data, with success rates separately for over 7 year old sub-population. Success rates are shown also for models trained with only >7 year olds. Latent space refers to data segments used in learning the latent representation, whereas fingerprinting task refers to segments projected to latent space that fingerprinting is then performed on.
