# Supplementary material for: Capturing individual variation in children’s electroencephalograms during nREM sleep
Source: PLoS Comput Biol. 2026 Jan 30;22(1):e1013931. doi: 10.1371/journal.pcbi.1013931 (PMC12885382; doi:10.1371/journal.pcbi.1013931)
Supplement: S7 Table — Used N1a+N2b data segments. Significant effects are marked with an asterisk: ***)p<0.001, **)p<0.01, *)p<0.05. (PDF) [file pcbi.1013931.s007.pdf]

Table S7. Regression results for BRRR and Correlational differentiability scores from mix of N1 and N2 sleep

	BRRR					Correlation				
	Coef.	SE	t	CI [0.025	0.975]	Coef.	SE	t	CI [0.025	0.975]
Intercept	1.34	0.06	20.65***	1.21	1.47	1.05	0.07	15.87***	0.92	1.18
noise	-0.02	0.29	-0.06	-0.59	0.56	0.03	0.25	0.13	-0.45	0.51
sex[M]	0.01	0.08	0.15	-0.14	0.17	-0.01	0.08	-0.16	-0.17	0.15
age	0.02	0.01	1.07	-0.01	0.04	0.01	0.02	0.87	-0.02	0.04
cap[FT]	-0.19	0.14	-1.36	-0.45	0.08	-0.21	0.14	-1.48	-0.49	0.07
noise:sex[M]	0.20	0.49	0.41	-0.76	1.16	0.19	0.41	0.45	-0.62	0.99
**Model Statistics**										
Residual Std. Error	0.974					0.977				
Multiple R^2	0.003					0.005				
Adj. R^2	-0.004					-0.002				
F-statistic (5,706)	0.49, $p = 0.782$					0.72, $p = 0.606$				

Used $N1_a + N2_b$ data segments. Significant effects are marked with an asterisk: ***)$p < 0.001$, **)$p < 0.01$, *)$p < 0.05$
