# Supplementary figures and images for: Capturing individual variation in children’s electroencephalograms during nREM sleep
Source: PLoS Comput Biol. 2026 Jan 30;22(1):e1013931. doi: 10.1371/journal.pcbi.1013931 (PMC12885382; doi:10.1371/journal.pcbi.1013931)

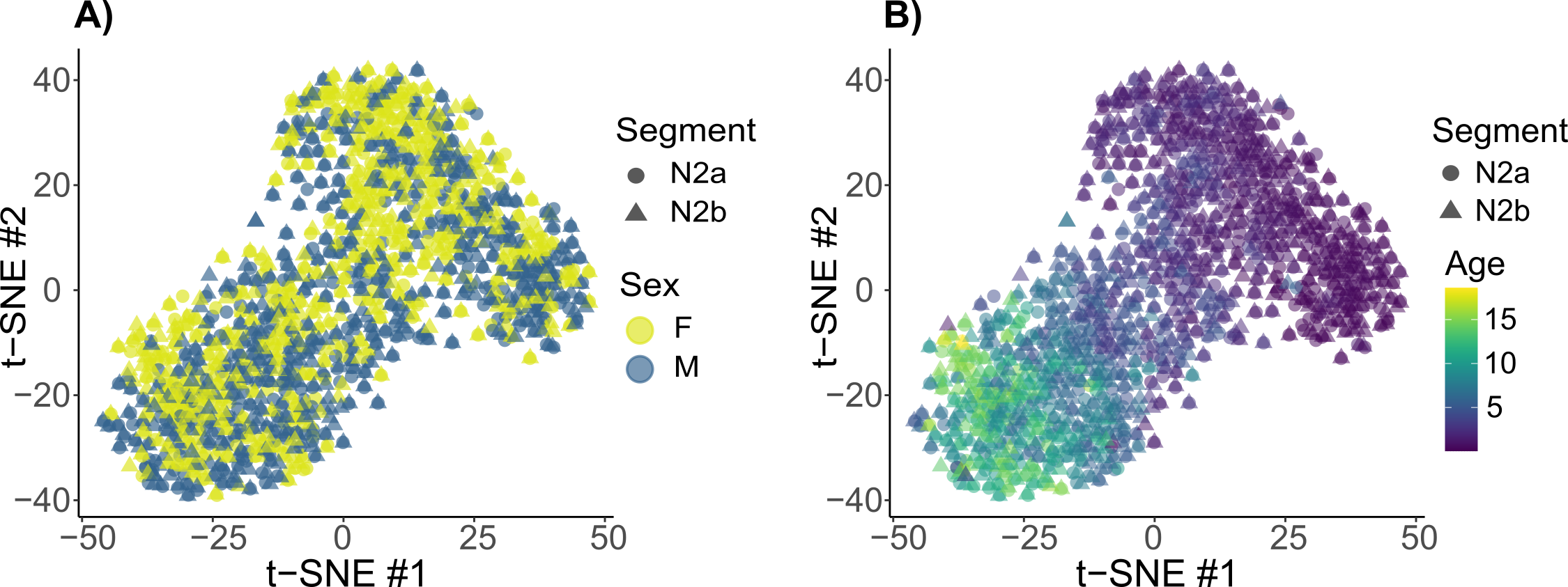

Supplement: S1 Fig — Two-dimensional t-SNE projection of the subjects projected on 30-dimensional latent mapping provided by BRRR (trained with N2 data). The first N2 segment is marked with a circle, the second with a triangle. As in Fig 5, we observe an age effect but not sex effect in the latent space. (TIFF) [file pcbi.1013931.s008.tiff]

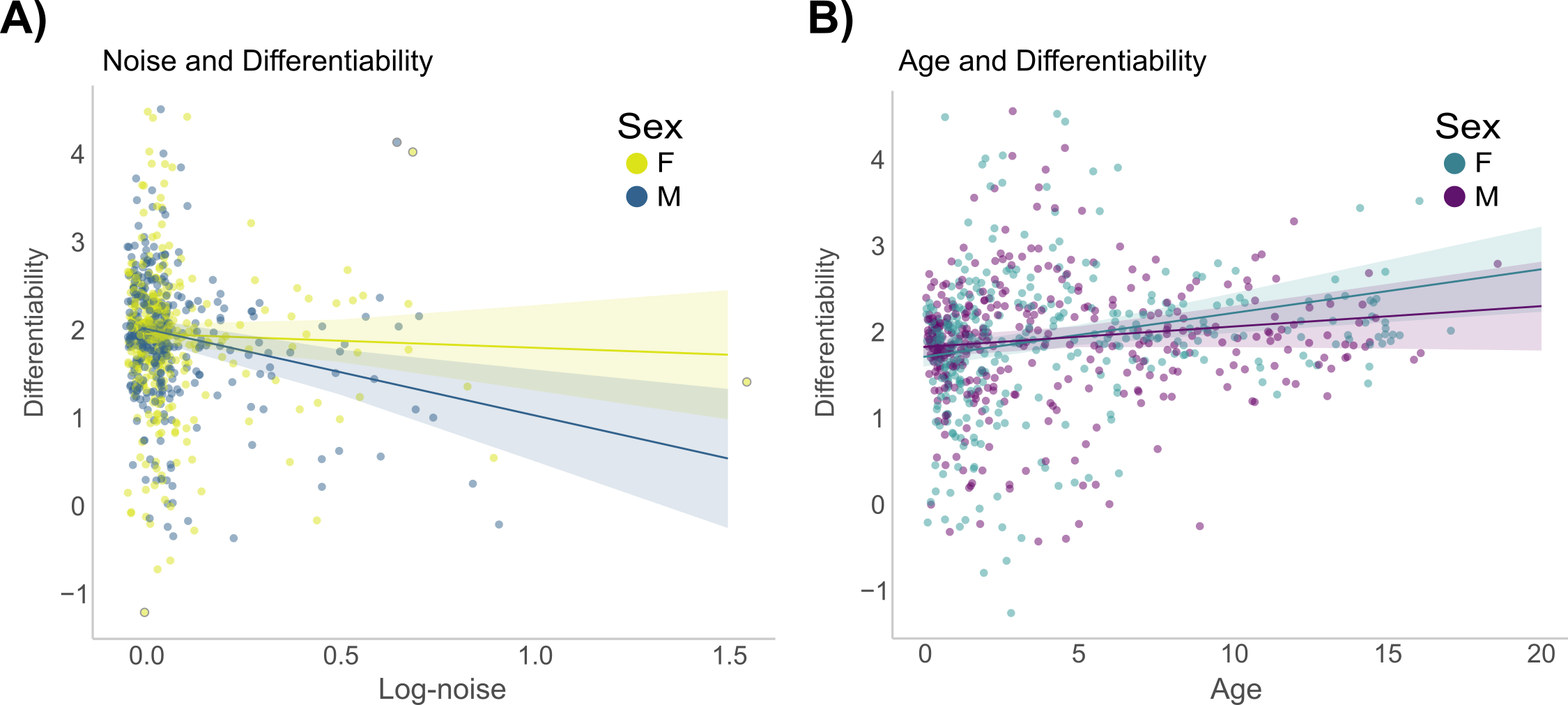

Supplement: S2 Fig — The differentiability and artifact-sex interaction effect is illustrated on the left (A: βFemale=−0.16,βMale=−0.99), B: the marginal age effect on differentiability (βFemale=0.05,βMale=0.02). (TIFF) [file pcbi.1013931.s009.tiff]

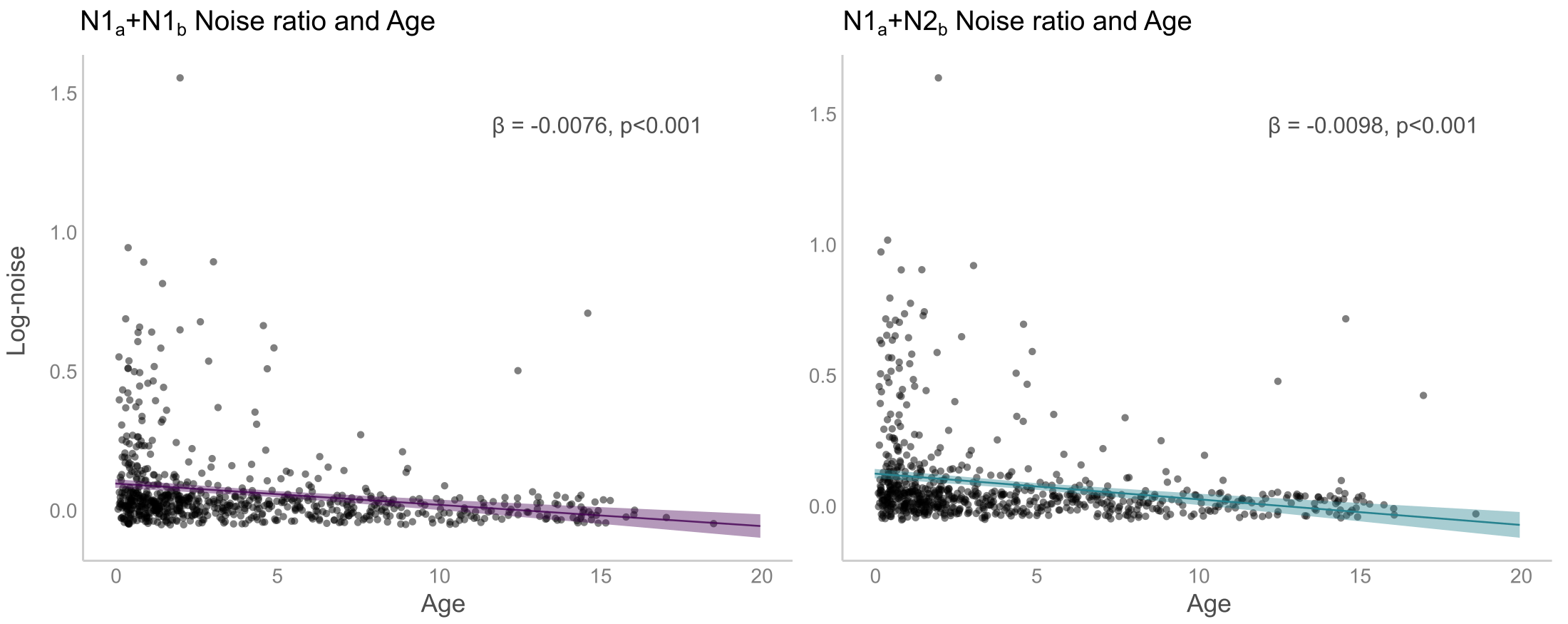

Supplement: S3 Fig — Left figure contains results for N1 sleep (β=−0.0076,p<0.001,r2=0.045), right for mix of N1 and N2 sleep (β=−0.0098,p<0.001,r2=0.054). (TIFF) [file pcbi.1013931.s010.tiff]

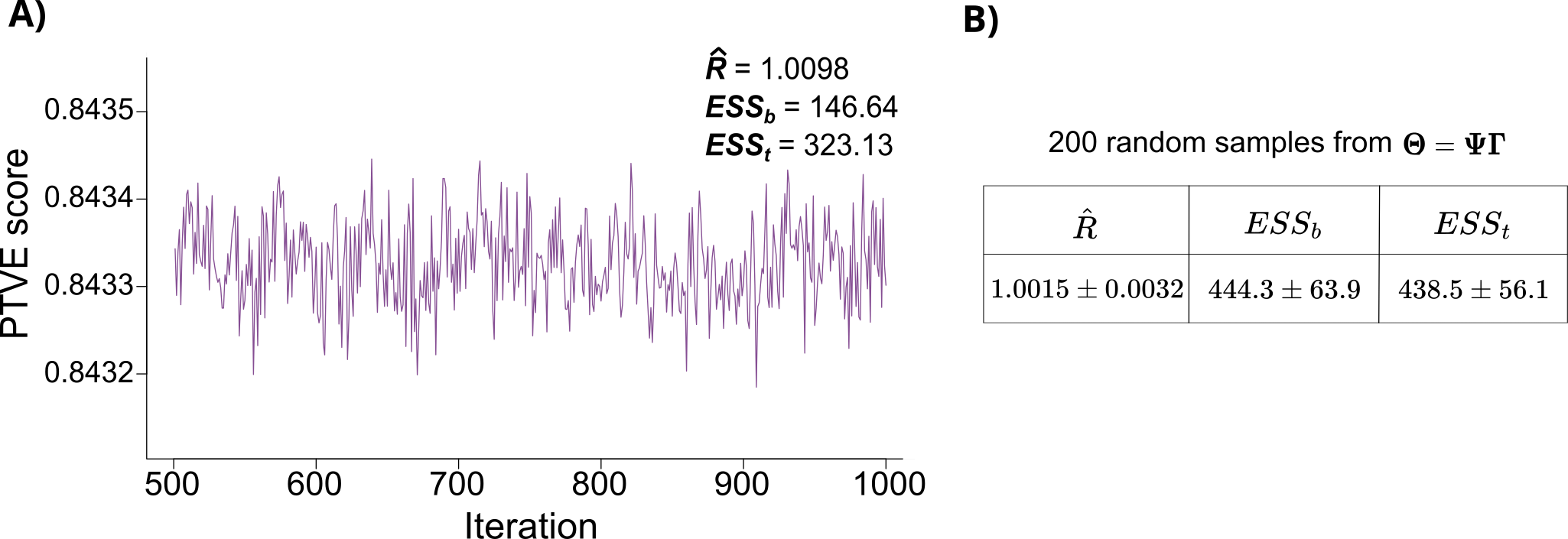

Supplement: S4 Fig — Fig A depicts a trace plot of the model PTVE across iterations (after discarding the first half of the samples as burn-in period) along with Markov chain convergence diagnostics, demonstrating good convergence. Table B summarizes the converge diagnostics (mean ± SD) of 200 randomly sampled indices from the low-rank regression coefficient matrix. (TIFF) [file pcbi.1013931.s011.tiff]
