# Supplementary material for: Capturing individual variation in children’s electroencephalograms during nREM sleep
Source: PLoS Comput Biol. 2026 Jan 30;22(1):e1013931. doi: 10.1371/journal.pcbi.1013931 (PMC12885382; doi:10.1371/journal.pcbi.1013931)
Supplement: S6 Table — Here N2 sleep segments are N2a+N2b. Significant effects are marked with an asterisk: ***)p<0.001, **)p<0.01, *)p<0.05. (PDF) [file pcbi.1013931.s006.pdf]

Table S6. Regression results for BRRR and Correlational differentiability scores from N2 sleep.

	BRRR					Correlation				
	Coef.	SE	t	CI [0.025	0.975]	Coef.	SE	t	CI [0.025	0.975]
Intercept	3.03	0.07	46.44***	2.90	3.16	2.25	0.04	56.11***	2.17	2.33
noise	-1.33	1.20	-1.11	-3.69	1.03	-0.54	0.29	-1.83	-1.11	0.03
sex[M]	-0.05	0.07	-0.65	-0.18	0.09	0.03	0.05	0.64	-0.06	0.12
age	-0.00	0.02	-0.05	-0.04	0.04	0.01	0.01	0.98	-0.01	0.03
cap[FT]	-0.15	0.14	-1.06	-0.43	0.13	-0.27	0.09	-2.94**	-0.44	-0.09
noise:sex[M]	0.60	1.36	0.44	-2.07	3.27	-0.01	0.37	-0.02	-0.73	0.72
**Model Statistics**										
Residual Std. Error	0.889					0.601				
Multiple R^2	0.025					0.032				
Adj. R^2	0.018					0.025				
F-statistic (5,706)	3.55, $p = 0.0036$					4.66, $p = 0.00034$				

Here N2 sleep segments are $N2_a + N2_b$. Significant effects are marked with an asterisk: ***)$p < 0.001$, **)$p < 0.01$, *)$p < 0.05$.
