# Supplementary material for: Capturing individual variation in children’s electroencephalograms during nREM sleep
Source: PLoS Comput Biol. 2026 Jan 30;22(1):e1013931. doi: 10.1371/journal.pcbi.1013931 (PMC12885382; doi:10.1371/journal.pcbi.1013931)
Supplement: Attachment — Submitted filename: Review comments.pdf [file pcbi.1013931.s012.pdf]

Review Comments

Black text: reviewer's comments

Cursive: citations from the manuscript

Blue text: our answers

Emphasized blue cursive: changes in the manuscript

Dear reviewing editor,

We thank you for the valuable feedback on our manuscript that has greatly helped to improve our manuscript. The result section is now extensively reorganized, and we have made new figures (4 & 6) to better address the reviewers' suggestions. In addition, we have re-checked our results against physiological artefacts and expanded the Discussion. We believe that we have been able to address all the questions and concerns of the reviewers. Detailed answers to reviewers' comments can be found below.

We hope that after these changes our manuscript can be accepted for publication in *PLOS computational biology*.

On behalf of all authors,

Verna Heikkinen

Major (Reviewer 1)

1. The literature review lacks any reference to previous developmental brain-fingerprinting studies. There has been considerable work examining how the stability of functional connectomes throughout development may serve as a biomarker for health, specifically mental health. See for example (Fu et al., 2023; Kaufmann et al., 2017, 2018). I believe that the paper would benefit from contextualizing the current findings with respect to broader brain-fingerprinting literature. This will help frame the paper and explain its unique contribution to literature.

We agree that our literature review on earlier developmental findings was not sufficient. We also thank the reviewer for the helpful reference suggestions that helped to frame the paper with respect to previous developmental fingerprinting studies.

We have now added several references to developmental brain-fingerprinting studies to Introduction: (page 3, lines 24-30): "***Compared with adults, children and adolescents exhibit higher intra-subject variability and consequently reduced distinctiveness in their connectome profiles (**Kaufmann et al., 2017; Fu et al., 2023**) and oscillatory activity (**da Silva-Castanheira et al., 2024**), a pattern that may stem from increased neuroplasticity (**Fu et al., 2023**). A delay in reaching adult-like distinctiveness of the connectome can be indicative of poorer mental health (**Kaufmann et al. 2017**) or psychiatric disorders, including schizophrenia (**Kaufmann et al. 2018**). As such, stability of brain fingerprints may be a useful metric when studying normal and pathological neurodevelopment (**Kaufmann et al. 2017, Fu et al. 2023**)".*** We have also expanded the Discussion in relation to these studies (E.g., page 22, lines 538-542).

We believe our work complements the existing literature by examining fingerprinting on large clinical EEG data sets. As applications aimed for clinical use should be tested with clinical data (Tapani et al. 2022), we demonstrate the applicability of latent-noise BRRR fingerprinting to low-density EEG that is feasible to collect in clinical settings. We have further clarified this in the Discussion (pages 22-23, lines 546-569).

2. The authors note in the introduction that: *"Previous approaches to find individually stable features from functional neuroimaging data have generally relied on correlations between different measurements of the same participants. While correlation approaches can reliably identify individuals within specific subject groups, such as patients and controls (17), it has remained uncertain which factors contribute to the differentiability of any individual subject."*
It is unclear to me whether they are referring to salient features for brain-fingerprinting or to specific demographic factors that may alter the brain-fingerprint of individuals. Could the authors clarify this point? In either case, I would argue that the correlational brain-fingerprint methodology can capture both features using group-level statistics. Overall, I am unclear about the specific advantage of the BRRR approach. Both approaches can derive the specific features which drive participant differentiation, albeit in different ways. Take, for example, their age-self-similarity effect in the latent space. Could the authors show that this effect is best captured by the BRRR approach over correlational brain-fingerprinting methods?

Regarding the first question, we agree with the reviewer that this part of the text was vague and somewhat misleading. We tried here to refer to our specific goal, i.e., testing our model's generalizability over sleep phases where we believe BRRR approach can be of benefit and expand the possibilities of fingerprinting approaches. We have now clarified this part in the Introduction (***"While correlation-based fingerprints are typically derived within one physiological state using all available data, we sought***

to determine whether reduced-rank regression could improve performance in more challenging fingerprinting scenarios —specifically across sleep stages and in refining individual feature estimation in noisy clinical settings.”, page 5 lines 79-83).

We have now analyzed the age-differentiability effect also with correlational methods and have included these results in the present manuscript (section **“Cortical fingerprints in children can be identified from EEG spectral power”** from page 7 onwards, current **Figures 4 & 6, and Supplementary Figure 2**). The BRRR approach appears to refine certain effects: For example, in N1 sleep, the age relationship is statistically significant with both methods, but the effect appears somewhat stronger with BRRR (β=0.11) than with the correlation method (β=0.05).

Previous studies have demonstrated that BRRR is suitable for connectivity-based fingerprinting (Haakana et al. 2024), illustrating the wide applicability of the method. As BRRR is a model, it can also be trained on one set of subjects and applied to another dataset: This can, e.g., help to assess the consistency of neurofunctional interindividual differences across datasets. We have stressed these points in the Discussion (page 20, lines 467-471).

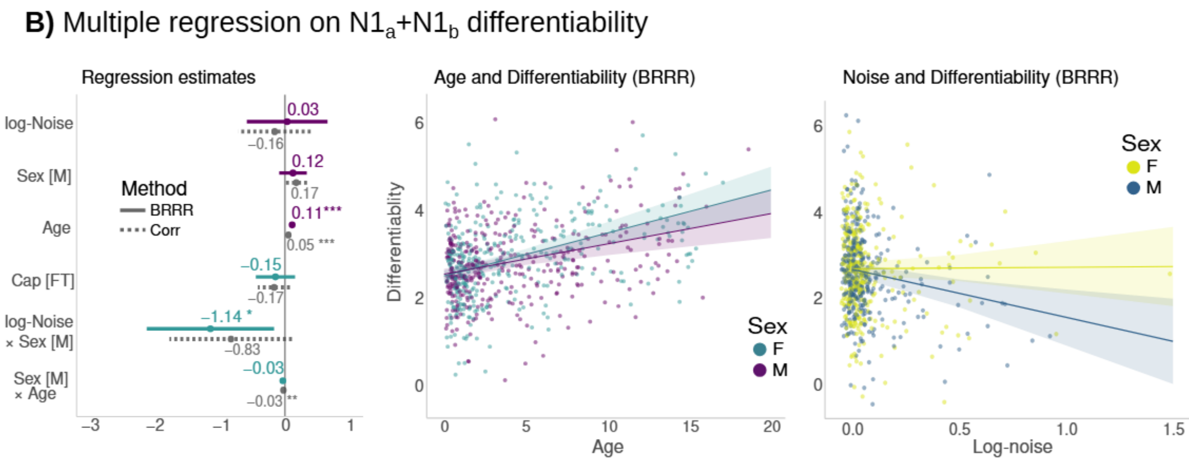

Current **Figure 6B.** Regression estimates for N1 differentiability scores for BRRR and correlation (left). The marginal age effect (middle) and noise (muscle artifact)-sex interaction (right) on differentiability with 95% confidence intervals are visualized for BRRR only. The statistically significant differences are marked with asterisks: (***) p <0.001, **) p < 0.01, *) p < 0.05).

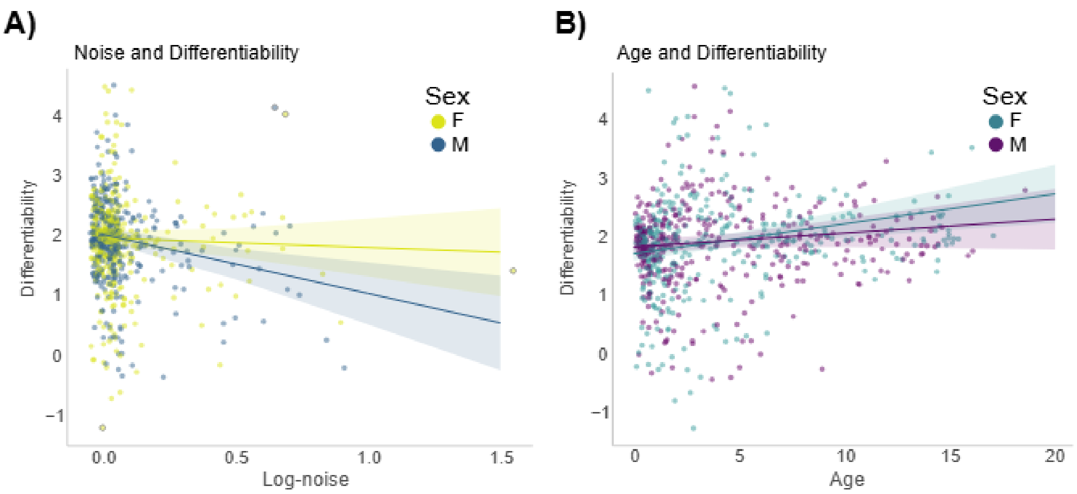

Figure S2. Visualizations of multiple regression on N1 ($N1_a + N1_b$) differentiability scores for correlation. The differentiability and artifact-sex interaction effect is illustrated on the left (**A**: $\beta_{\text{Female}} = -0.16, \beta_{\text{Male}} = -0.99$), **B**: the marginal age effect on differentiability ($\beta_{\text{Female}} = 0.05, \beta_{\text{Male}} = 0.02$)

3. How do you interpret the differences in stability of the spectra between sleep stages (Figure 2) concerning neurodevelopmental effects? The point the authors are trying to make is unclear to me. From the introduction, I would have thought the authors would explore an age-by-sleep-stage interaction on the stability of PSD estimates. Yet, they report that most of the age groups show differences in the stability of spectra across sleep stages. Therefore, I am unclear how the authors interpret the findings of Figure 2. I interpret these findings to suggest that high-frequency bands are unstable between sleep stages for all age groups.

We apologize for the previous unclarities in the manuscript. In **Figure 2**, we wanted to show the real clinical data, with all its possible confounds that might disturb the later analysis approaches, without any statements about the replicability of the effects. Despite the obvious variability in the data segments within sleep stages, especially at the frequencies above 25 Hz, our modelling approach succeeded in fingerprinting the data with good accuracy in all age groups. We argue that especially in the presence of PSD instabilities, fingerprinting even within a sleep stage is not trivial, and that especially correlation-based measures might suffer from this variability: we discuss this now in the manuscript (page 7, lines 131-135).

On a related note, the authors should specify how they dealt with regional differences in the power spectra. Did they fit this model for the average spectra across all electrodes?

Although **Figure 2** shows cluster permutation results calculated for the grand averages over all electrodes, the BRRR model was fit without any averaging over the electrodes. To account for varying power levels across channels, we used relative power, i.e., scaled the PSDs by dividing them by the total power of each channel. All the remaining analysis was done with the relative power estimates. We have clarified this in the text (page 7, lines 139-142: ***"We first calculated the bandpower over 13 logarithmically widening frequency bands between 1 and 43 Hz separately for each channel (see Figure 3A-B). The channel-wise relative bandpowers in each frequency band were then used as a feature in the BRRR algorithm."***)

4. The methodology of the 'null models' is unclear to me. Based on the correlational brain-fingerprinting method, no model is trained. The similarity between two data segments/ recordings are analyzed; therefore, it is unclear how the model is validated on an 'out-of-sample'. Moreover, it is unclear how one can fingerprint with more than one recording/segment. Overall, the methodology of Figure 4 is very unclear to me. I believe the paper and the interpretability of the results would be greatly improved if the authors clarified this point.

We apologize for the unclear explanation of the model in the previous draft. The term 'Out-of-sample' -validation was used to refer to cases where we used latent space trained with certain segments in fingerprinting on other segments. As the term evokes an idea of a separate data set, which we did not have, we have removed the term and instead describe this idea in following terms (page 8, lines 168-176): ***"We also calculated the success rates by using all the observations in different data segment combinations. To examine the stability of the EEG fingerprint over the course of the sleep recording, we tested how well the latent representation of the BRRR model generalizes to subsequent PSD segments of the same individuals. Ideally, the model should assign each new observation to the correct individual by preserving short within-subject distances in the latent space learned from prior data. For this, we used an additional validation procedure, where the distances between subjects were calculated using additional PSD segments of N1 or N2 sleep, projected into the latent space."***

We have clarified the validation procedure also in Methods and removed the 'null model' term that referred to correlation-based fingerprinting (see, e.g., page 5 lines 88-89). We have also thoroughly revised **Figure 4** (see below) and Supplementary tables: we now compare BRRR fingerprinting to correlation-based fingerprinting in cases where such a comparison is warranted (**Supplementary Table S4**).

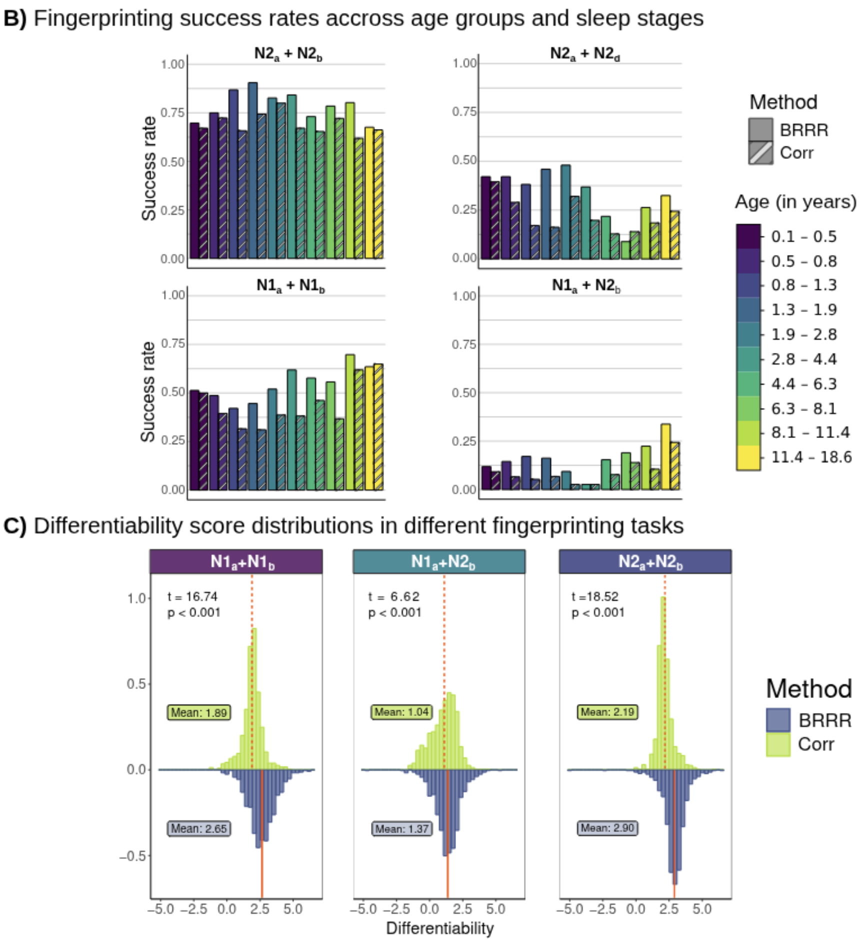

Figure 4. Success rates and differentiability scores across different models and subject groups. **A**: Averaged 10-fold cross-validation success rates for BRRR models using 2 or 3 training data segments. Success rates are shown separately for differentiating subjects in groups 'All' and '>7-year-olds'. **B**: Fingerprinting success rate on all data per age group, for both BRRR and correlation. Upper row: fingerprinting within N2 sleep, either using subsequent segments (left) or segments further apart in time (right). Bottom row: fingerprinting results within N1 sleep (left) and between sleep sages (right) **C**: differentiability score distributions in three fingerprinting tasks for BRRR and correlation -based fingerprinting. The means of the differentiability score distributions are indicated by vertical lines and compared using t-test.

5. How does the self-similarity effect compare to traditional brain-fingerprinting measures (i.e., self-similarity as measured by correlation)? I believe that the inclusion of these analyses would make the results easier to interpret.

I also want to note that the decreasing self-similarity with age is the opposite prediction of the brain-fingerprint literature (Fu et al., 2023; Kaufmann et al., 2017, 2018). Similarly, it seemingly contradicts the authors' own predictions, *"model trained using older participants could provide more stable fingerprint"* and *"sleep patterns would become increasingly stable with age"*. These findings therefore, are very difficult to interpret without a benchmark from more traditional self-similarity measures. Is the model introducing an age bias, or is it something specific to sleep stages or the dataset itself? Could the authors explicitly state why they believe the results are in the opposite direction of their hypothesis and the literature.

We now notice that our previous nomenclature was indeed poor and misleading. As BRRR is a distance-based measure, the 'self-similarity' was in fact self-distance, which produces an opposing trend compared to the correlation-based similarity measure: here a small self-distance refers to good differentiability. We apologize for our carelessness with the terminology which caused confusion in the previous draft. We have now changed the terms to better fit the existing fingerprinting literature: we define Z-scored differentiability (instead of self-similarity) for both BRRR and correlation methods, to ensure better comparability and similar direction of effects (page 8, lines 180-183: ***"For both methods, we defined the differentiability as a Z-scored self-distance (or correlation) against the distances (correlations) to others. The Z-scoring approach is described in the Methods section (Eq. 3, Statistical testing)"***).

Both correlation-based fingerprint and BRRR show similar trends: fingerprinting accuracy improves with age when fingerprinting is conducted within the N1 sleep stage, and the age effect appears to be stronger with BRRR. We did not observe any significant age relationships with this differentiability metric in other sleep stages. As described in the answer to point 2, we now also include the correlational differentiability measure in the result section (e.g., **Table 1**, **Figures 4C & 6,** presented also above).

	BRRR					Correlation				
	Coef.	SE	t	CI [0.025	0.975]	Coef.	SE	t	CI [0.025	0.975]
Intercept	2.27	0.07	30.73***	2.12	2.41	1.75	0.06	27.26***	1.63	1.87
noise	0.03	0.32	0.11	-0.58	0.65	-0.16	0.28	-0.57	-0.71	0.39
sex[M]	0.12	0.11	1.14	-0.09	0.33	0.17	0.09	1.96	0.00	0.34
age	0.11	0.02	5.89***	0.07	0.15	0.05	0.01	3.76***	0.02	0.08
cap[FT]	-0.15	0.15	-0.95	-0.45	0.16	-0.17	0.13	-1.34	-0.42	0.08
noise:sex[M]	-1.14	0.50	-2.30*	-2.12	-0.17	-0.83	0.48	-1.74	-1.77	0.11
sex[M]:age	-0.03	0.02	-1.73	-0.07	0.00	-0.03	0.01	-2.58**	-0.05	-0.01
**Model Statistics**										
Residual Std. Error	0.919					0.742				
Multiple R^2	0.143					0.042				
Adj. R^2	0.136					0.034				
F-statistic (6,705)	19.68, $p = 2.6 \times 10^{-21}$					5.19, $p = 3.1 \times 10^{-5}$				

Table 1. Regression results for BRRR and Correlational differentiability scores from N1 sleep ($N1_a + N1_b$). Significant effects are marked with an asterisk: ***)$p < 0.001$, **)$p < 0.01$, *)$p < 0.05$

6. The results section could be reorganized. The panels of each of the Figures are not discussed in order, which makes it difficult for the reader to follow the figures. For example, the section "BRRR fingerprints generalize better compared to correlation-based fingerprinting" which is about Figure 4, is presented after Figure 6.

We have now extensively reorganized the text and elaborated on the relevant parts in the Results section to clarify the methodology and the interpretation of the results. We have also changed **Figures 4** and **6.** Comparison to correlation-based fingerprinting is now carried throughout the Results section, to avoid discussing the Figures in disorder.

7. The authors note: *"Thus, a similarity metric based on just correlation does not appear to generalize across sleep stages and suffers from increased dimensionality and heterogeneity of the training data."*
One could argue that this lack of stability is very interesting and something to be understood and not reduced with the BRRR model. For example, previous literature reports that the brain-fingerprints of individuals with Parkinson's disease are less stable than age-matched controls (da Silva Castanheira, Wiesman, Hansen, et al., 2024; Troisi Lopez et al., 2023). This is specifically driven by arrhythmic brain activity. I believe that exploring the instability of brain activity is a useful approach to understanding why participants are less differentiable from the brain activity and may offer novel biological insights. Indeed, the authors themselves highlight this in the introduction: *"The stability of these individual features, or the lack thereof, could similarly serve as a surrogate biomarker of risk of atypical development."*
I am not saying that there is no added value of the BRRR approach, but I believe that the authors can do a better job at contextualizing how and when the BRRR approach is useful while also highlighting some advantages of the correlational brain-fingerprinting methods approach.

The point the reviewer raises here is indeed highly relevant, and we thank the reviewer for the valuable suggestions. We have now conducted the full analysis using both fingerprinting methods and dedicated a chapter in the Discussion to address the advantages and weaknesses of both methods (***Correlation and latent noise approaches in modeling individual differences***, pages 19-21, lines 447-518). We believe that BRRR can provide additional information beyond correlation-based approaches particularly in cases where data quality varies—as is often the case in large datasets, where correlation methods tend to show reduced fingerprinting accuracy. BRRR only uses those features that are informative for individuality, but as the model performance depends on the latent space dimension, which is a hyperparameter, it needs to be set separately for different applications. At the same time, correlation-based fingerprints are more readily interpretable and computationally easier to obtain; correlation could also be used as a similarity metric in the BRRR latent space. BRRR and correlation-based fingerprinting thus appear complementary approaches: both methods find similar main trends in our data with slightly different relationships between population and artifact confounders, giving further support to the neurophysiological interpretations on our data.

We note that ***"correlational fingerprinting is computationally simpler and does not require hyperparameter optimization, and the method has given excellent results in the context of individual connectomes"*** (page 20, lines 465-467) and ***"While BRRR yields better results in terms of differentation, the structure of the latent space is stochastic [...] Dimensionality reduction methods in fingerprinting might introduce a trade-off between simplicity and performance"*** (lines 481-487).

We agree that the reduced stability of brain-fingerprints could be very useful both during the development, and again later in life as a marker of degenerative processes that "fade" the fingerprints.

8. More generally, I feel like a discussion on the utility of a BRRR brain-fingerprint model that can predict inter-individual differences across many physiological states would benefit the paper's structure. Sleep and wakefulness represent very different mental and physiological states, and as such, the functional connectivity of the brain and its dynamics are radically different. Should we expect to be able to differentiate individuals across these states based on functional activity? Could one not argue that the salient features for participant differentiation across various physiological states are driven by anatomical and other non-electrophysiological factors, which are unlikely to be altered by physiological states?

The reviewer brings up significant questions. In our present work, we demonstrate identifiability across sleep stages, despite the assumption that, e.g., artefacts such as eye blinks and movements are likely to be more abundant during N1 sleep and should affect the differentiation results negatively. We have previously shown (Leppäaho et al. 2019) that MEG-PSD components that differentiate between sibling pairs also differentiate between individuals, even across measurement tasks. Some of these components were associated with underlying genetic factors: thus they appear to be meaningful descriptors of functional brain activity despite not being altered by the physiological state. Interestingly, our recent study in the same sibling pairs demonstrated that different components of oscillatory somatomotor activity have variable heritability and thus likely reflect different underlying generation mechanisms, some depending on more fixed anatomical parameters and others reflecting dynamic functional characteristics (Pauls et al. 2024). On the basis of these results, we believe that the different electrophysiological features that drive the differentiation between individuals are likely to reflect both anatomical and functional factors that are not easy to separate from each other. We discuss this now also in the manuscript (page 18, lines 297-409).

We argue in favor of such generalizability especially in the context of BRRR: ***"The strength of the BRRR approach is highlighted when using different sleep segment to train and test the model: latent representation learned from mixed sleep stage generalized to other sleep segments,*** ***and had slightly higher success rates than the correlational approach. This is likely due to the low-dimensional representation of the data which reduces noise (including measurement noise and importantly, non-modeled effects) while refining the individual features. Thus, latent mapping can maintain low within-subject distances even when presented with new data."*** (page 21, lines 491-497). More generally, latent variable modeling can be utilized to synthetize complicated and noisy brain responses and behavioural or clinical data and model between-subject variability more reliably (Cooper et al. 2019).

9. In line with the point above, what steps did the authors take to address non-electrophysiological confounds?

Firstly, eye-movements, blinks and heartbeat artefact were removed algorithmically using independent component analysis, which is a common M/EEG pre-processing step (Fast-ICA, implemented in MNEpython; Described in Methods/EEG preprocessing, page 27 lines 665-671).

In the later analysis, to see whether movement/muscle artifacts or EEG cap size affected the results, we added multiple regression analysis (**Eq. 4** under Methods/Statistical testing) to see if these variables confounded the results (page 32, lines 819-828; for the results see **Figure 6** presented above and **Supplementary Tables S6 and S7** below). When fingerprinting on N1 sleep, increases in the log-ratio of muscle artifact were associated with larger reductions in the boys' than girls' BRRR-differentiability scores. During other fingerprinting tasks, we did not observe a significant relationship between muscle artifact ratios and differentiability. When fingerprinting on N2 sleep, correlation-based differentiability scores suffered from the larger cap size. Here, the cap size mediates the effect of age: N2 PSD estimates were unstable at alpha- or sleep spindle range oscillations for older participants measured with the larger cap size (typically utilized with subjects from 5-year-old onwards; **Figure 2**).

	BRRR					Correlation				
	Coef.	SE	t	CI [0.025	0.975]	Coef.	SE	t	CI [0.025	0.975]
Intercept	3.03	0.07	46.44***	2.90	3.16	2.25	0.04	56.11***	2.17	2.33
noise	-1.33	1.20	-1.11	-3.69	1.03	-0.54	0.29	-1.83	-1.11	0.03
sex[M]	-0.05	0.07	-0.65	-0.18	0.09	0.03	0.05	0.64	-0.06	0.12
age	-0.00	0.02	-0.05	-0.04	0.04	0.01	0.01	0.98	-0.01	0.03
cap[FT]	-0.15	0.14	-1.06	-0.43	0.13	-0.27	0.09	-2.94**	-0.44	-0.09
noise:sex[M]	0.60	1.36	0.44	-2.07	3.27	-0.01	0.37	-0.02	-0.73	0.72
**Model Statistics**										
Residual Std. Error			0.889					0.601		
Multiple R^2			0.025					0.032		
Adj. R^2			0.018					0.025		
F-statistic (5,706)			3.55, $p = 0.0036$					4.66, $p = 0.00034$		

Table S6. Regression results for BRRR and Correlational differentiability scores from N2 sleep ($N2_a + N2_b$). Significant effects are marked with an asterisk: ***)$p < 0.001$, **)$p < 0.01$, *)$p < 0.05$.

	BRRR					Correlation				
	Coef.	SE	t	CI [0.025	0.975]	Coef.	SE	t	CI [0.025	0.975]
Intercept	1.34	0.06	20.65***	1.21	1.47	1.05	0.07	15.87***	0.92	1.18
noise	-0.02	0.29	-0.06	-0.59	0.56	0.03	0.25	0.13	-0.45	0.51
sex[M]	0.01	0.08	0.15	-0.14	0.17	-0.01	0.08	-0.16	-0.17	0.15
age	0.02	0.01	1.07	-0.01	0.04	0.01	0.02	0.87	-0.02	0.04
cap[FT]	-0.19	0.14	-1.36	-0.45	0.08	-0.21	0.14	-1.48	-0.49	0.07
noise:sex[M]	0.20	0.49	0.41	-0.76	1.16	0.19	0.41	0.45	-0.62	0.99
**Model Statistics**										
Residual Std. Error			0.974					0.977		
Multiple R^2			0.003					0.005		
Adj. R^2			-0.004					-0.002		
F-statistic (5,706)			0.49, $p = 0.782$					0.72, $p = 0.606$		

Table S7. Regression results for BRRR and Correlational differentiability scores from mix of N1 sleep and N2 ($N1_a + N2_b$). Significant effects are marked with an asterisk: ***)$p < 0.001$, **)$p < 0.01$, *)$p < 0.05$

Minor (Reviewer 1)

1. The authors discuss how they believe sleep EEG is a great opportunity to study children, because of the inherent difficulty in collecting neural data from this population. I would appreciate it if the authors could discuss specific challenges of generalizing sleep EEG-derived fingerprints to wakefulness versus artifact correction on brain-fingerprints derived from awake individuals.

We thank for the good point raised by the reviewer. Generally, artifact correction appears to improve the BRRR fingerprinting accuracy even during wakefulness (own unpublished observations from MEG data in adults), and the effect is likely to be bigger for the more artifact-prone EEG recordings. We have now included our thoughts on this in the Discussion (page 23, lines 559-568: ***"In very young children, awake EEG can have a substantial amount of artifacts that considerably decrease the signal-to-noise ratio and complicate finding meaningful fingerprints. In our analysis, more heterogeneous input data (data from both sleep stages, or from segments further***

apart in the recording) produced more generalizable fingerprints. BRRR –based sleep fingerprints are thus likely to be generalizable to wakefulness as well, but the performance may depend on the states used to train the model. Furthermore, sleep contains oscillatory phenomena that are not present in wakefulness, which may reduce generalizability. Future studies could address this by using data from awake individuals, combined with efficient removal of the more abundant artifacts in the awake EEG.").

2. How did the authors test the non-linearity of the spectral AUC effect? To me, it seems like they ran a series of t-tests, but I believe a polynomial regression model would be a better test of the non-linear effect. On a more general note, I believe that including a sentence or two before each result explaining the methodology would be helpful to the reader. It would provide them with important context on which statistical test was performed.

We thank the reviewer for this very helpful suggestion: we have now replaced the series of t-tests with a polynomial regression model to demonstrate the non-linear AUC effect. The results are discussed in p. 6 (lines 110-116). We have also added a new **Figure 1C** (see below) and the regression results in **Supplementary Tables S1** (N1 AUC effect) and **S2** (N2 AUC effect). Furthermore, we have now elaborated on the reasoning of each statistical test in the text (E.g, page 6, line 125-126).

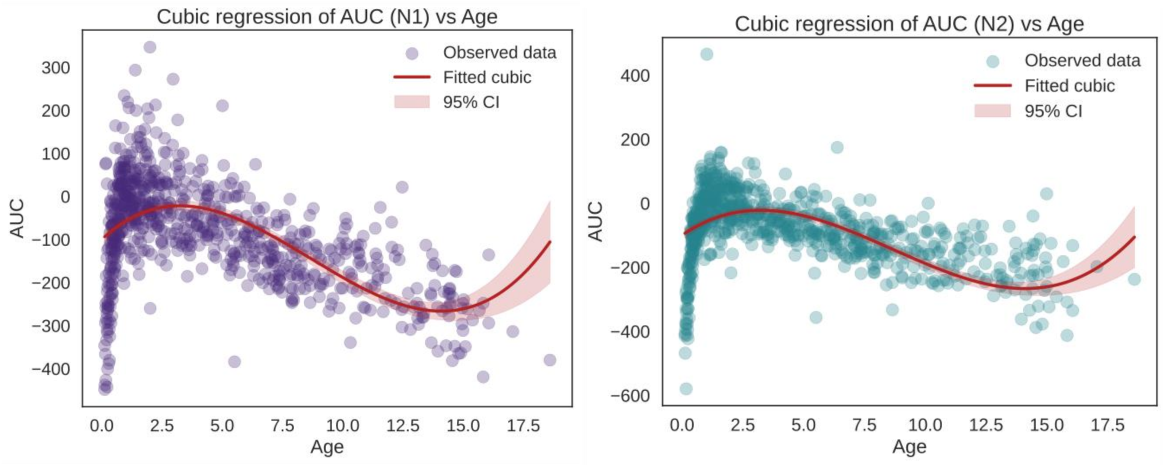

3. How do you ensure the placement of electrodes is comparable across age groups, given the very different head sizes with age?

The cap (size) was always selected and positioned by a highly experienced clinical neurophysiology nurse, and the positioning followed the standard positioning as accurately as possible. Still, irregular head shape of an infant may cause data loss, especially in electrodes towards the edges of the cap due to a bad fit. However, we consider this quite a minor problem: even if some of the data were of worse quality because of poorer fit, this should only affect our results negatively.

For future research, source-localized PSDs might alleviate this problem and allow spatial inferences about the oscillatory features affecting fingerprints. These, however, require MRIs. Head circumference measurements could be used as proxies to scale template MRIs, to inspect to which degree age-related changes in differentiability are attributable to head size. Such an approach was recently used for predicting functional brain age from pediatric sleep EEG, EEG activity significantly outperformed brain-age prediction based on head circumference (Iyer et al. 2024). We now discuss this in the manuscript (pages 24-25, lines 608-612).

4. I find the red lines in Figure 4 hard to read. I would recommend that the authors find a different way of depicting their results.

We have now reformatted **Figure 4** and provide the model fit metric values only in the **Supplementary Tables S3 & S4**.

5. The authors *"hypothesized that the model trained using older participants could provide more stable fingerprints compared to the model using all participants, especially across sleep stages."* Yet, it seems like spectral features are less stable for the older age group (Figure 2), i.e., more grey segments with age, especially for the N2. How do the authors reconcile these effects?

We find more stable fingerprints within the N1 sleep stage using older participants. The spectral features within N2 sleep seem indeed to be less stable in the older age groups, which is also reflected in **Figure 4B** (upper right). However, when fingerprinting across sleep stages, BRRR appears to find differentiating features in one sleep stage that are also differentiating in the other sleep stage in older subjects. We have changed the wording to better reflect this (page 7, lines 147-148: ***"We hypothesized that the model trained using older participants could provide more stable across sleep stage fingerprints compared to the model based on all participants."***).

6. For consistency, I would flip the color bar of Figure 3d such that the colors in both matrices correspond to the same thing (i.e., hot colors mean more similar).

We have flipped the color bar as suggested. It indeed looks better this way.

7. The name self-distance is misleading in Figure 4c and the subsequent analyses. The authors define a z-score-like metric of distance in the latent space, not the absolute distance between a participant's two recordings. Therefore, when looking at the difference between self-similarity and between-subjects similarity, we observe large differences, but this comparison is not fair as the metrics are on different scales (one being an absolute measure of distance and the other relative). I would recommend that the authors compute the 'raw' self-similarity distance metric for their analyses or rename this metric to something akin to differentiability (which I believe it more closely measures).

We agree that the nomenclature in the previous draft was subpar. As detailed above (major revision point 5), we have now defined a Z-scored differentiability measure, akin to the one used in previous research, and employ it throughout the text. We have also removed between-subject similarity figures (see **Figure 4** above) and only use the relative Z-scored differentiability metric in the analysis.

8. The authors speculate that the effects observed in the low-dimensional space might be driven by arrhythmic features, but this is precisely what traditional brain fingerprinting approaches would be able to tell you (da Silva Castanheira, Wiesman, Hansen, et al., 2024; da Silva Castanheira, Wiesman, Taylor, et al., 2024).

How does the BRRR model improve interpretability? I think the authors convincingly show that the BRRR method improves accuracy, but they do little to examine how it impacts the salient features for participant differentiation (which one may argue is easier to do on brain-fingerprints not defined in a latent space). Again, I want to emphasize that I believe that the BRRR method has specific advantages, but these are not immediately evident when reading the paper and can be made more obvious. Perhaps a thorough discussion of the advantages of each technique and their potential application would help clarify this point.

We thank the reviewer for raising this point and helpful references. The result of aperiodic activity being salient in individual fingerprinting is now included also in the discussion (page 18, lines 422-424: ***"It is therefore possible that the age-effects observed in the low-dimensional space reflect changes particularly in the aperiodic power, a result that has been observed in fingerprinting studies before (****da Silva Castanheira et al. 2024****)"***). Furthermore, we have now restructured the Discussion to consider both fingerprinting methods and have dedicated a chapter for this (**Correlation and latent noise approaches in modeling individual differences**, pages 19-21, lines 447-518). See also our reply to the major revision point 8.

References

da Silva Castanheira, J., Wiesman, A. I., Hansen, J. Y., Misic, B., Baillet, S., Breitner, J., Poirier, J., Bellec, P., Bohbot, V., & Chakravarty, M. (2024). The neurophysiological brain-fingerprint of Parkinson's disease. EBioMedicine, 105. https://www.thelancet.com/journals/ebiom/article/PIIS2352-3964(24)00236-6/fulltext

da Silva Castanheira, J., Wiesman, A. I., Taylor, M. J., & Baillet, S. (2024). The Lifespan Evolution of Individualized Neurophysiological Traits. bioRxiv: The Preprint Server for Biology, 2024.11.27.624077. https://doi.org/10.1101/2024.11.27.624077

Fu, Z., Liu, J., Salman, M. S., Sui, J., & Calhoun, V. D. (2023). Functional connectivity uniqueness and variability? Linkages with cognitive and psychiatric problems in children. Nature Mental Health, 1(12), Article 12. https://doi.org/10.1038/s44220-023-00151-8

Kaufmann, T., Alnæs, D., Brandt, C. L., Bettella, F., Djurovic, S., Andreassen, O. A., & Westlye, L. T. (2018). Stability of the Brain Functional Connectome Fingerprint in Individuals With Schizophrenia. JAMA Psychiatry, 75(7), 749–751. https://doi.org/10.1001/jamapsychiatry.2018.0844

Kaufmann, T., Alnæs, D., Doan, N. T., Brandt, C. L., Andreassen, O. A., & Westlye, L. T. (2017). Delayed stabilization and individualization in connectome development are related to psychiatric disorders. Nature Neuroscience, 20(4), Article 4. https://doi.org/10.1038/nn.4511

Troisi Lopez, E., Minino, R., Liparoti, M., Polverino, A., Romano, A., De Micco, R., Lucidi, F., Tessitore, A., Amico, E., Sorrentino, G., Jirsa, V., & Sorrentino, P. (2023). Fading of brain network fingerprint in Parkinson's disease predicts motor clinical impairment. Human Brain Mapping, 44(3), 1239–1250. https://doi.org/10.1002/hbm.26156

Reviewer 2 Comments

1. BRRR MODEL DEFINITION

The dimensionality of the latent space is fixed to k = 30. The definition of this parameter appears arbitrary. How was this value chosen?

We apologize for the unclarity in the previous manuscript regarding the latent space dimension parameter choice. We have now clarified the selection criteria that was based on the elbow-method in the text (page 8, lines 160-161 in Results and page 30, lines 765-782 in Methods: ***"Effects of feature and observation space sizes"***. See also **Figure 8**). K=30 was chosen as a point after which model accuracy and fit, measured by PTVE, did not improve rapidly. Similar reasoning behind the latent space dimension parameter choice were used by Haakana et al. 2024.

2. EFFECT OF AGE ON FINGERPRINTING ACCURACY

A substantial part of this work's claims relies on the increasing stability of individual neural fingerprints with age. However, these results stem from the comparison of a model fitted to the entire cohort with a second model fitted only participants older than 7. First, the definition of the older group appears subjective, particularly as it does not match the age groups used in the first analyses. What is the rationale supporting a clear split from 7 years onwards? Next, comparing the performance of the model trained with all ages to one trained with only participants closer in age might be biased, as more subtle features could become of importance in the latter case. How does the accuracy compare for the >7 y.o. between both models?

To provide a stronger support to this hypothesis, it would be of interest to show the recognition accuracy by age group, similarly to Figure 4A/B.

We thank the reviewer for these important remarks and useful suggestions. The split at 7 years of age was motivated by the start of formal education (which is at 7 in Finland). We have included this detail in the text (Results, page 7 line 145). We have revised the results to include the accuracy results per age group (**Figure 4B**) to better fit the first analyses. We describe these results and compare them to a model fitted to older participants (page 11, lines 218-227: ***"When examining the success rates per age group, we found different patterns depending on the fingerprinting task (Figure 4B). Within subsequent segments of N2 sleep (upper left), the success rates were high***

in all age groups but seemed to increase by age in N1 sleep and mixed sleep stage (N1a+N2b) fingerprinting (bottom row), corresponding to results for over 7-year-olds in Figure 4A. When fingerprinting on far-apart N2 segments, the older age groups did not appear to be better differentiated from the rest. Here again BRRR and correlation show similar trends, except for the N2a+N2d fingerprinting task, where BRRR achieves highest success rates for children aged 1.3-2.8 years and correlation performs best in under 6-month-olds”).

Furthermore, we added the results of over 7-year olds in models trained with all data (**Supplementary Table S5**, see also below) and compare them to models trained with only this population (page 12, lines 249-255: ***“We also examined the success rates separately for over seven year olds in the models trained using all data (Table S5). The success rates were always higher than in the whole population, but when trying to generalize from N2 to N1 sleep stage, the success rates were lower (0.58-0.63) compared to models trained exclusively with over 7-year-olds. Here, restricting the analysis to older participants may allow the model to find more generalizable features.”***). Typically, the models trained only with >7 y.o. did not outperform the sub-population of >7 y.o. compared to models trained with all data; therefore we believe that models trained with only participants close to each other in age does not introduce any positive bias.

		All data		>7 y.o
**Latent space**	**Fingerprinting task**	**SR**(All)	**SR**(>7 y.o.)	**SR**
$N2_a$+$N2_d$	$N2_b$+$N2_c$	0.80	0.89	0.76
$N1_a$+$N2_d$	$N2_a$+$N2_b$	0.74	0.82	0.72
$N2_a$+$N2_b$	$N2_c$+$N2_d$	0.74	0.82	0.69
$N1_a$+$N1_b$	$N2_a$+$N2_b$	0.71	0.80	0.69
$N2_a$+$N2_d$	$N1_a$+$N1_b$	0.54	0.63	0.76
$N2_a$+$N2_b$	$N1_a$+$N1_b$	0.48	0.58	0.72
$N1_a$+$N1_b$	$N2_a$+$N2_d$	0.19	0.29	0.25
$N1_a$+$N2_b$	$N1_b$+$N2_a$	0.27	0.42	0.41
$N2_c$+$N2_d$	$N1_a$+$N2_a$	0.11	0.15	0.25

Table S5. Summary of the generalizability of different BRRR models ($K = 30$), ordered by descending success rates on all data. Results are shown for models trained with all data, with success rates separately for over 7 year olds sub-population. Success rates are shown also for models trained with only >7 year olds. Latent space refers to data segments used in learning the latent representation, whereas fingerprinting task refers to segments projected to latent space that fingerprinting is then performed on.

3. DATA QUALITY AND EEG PREPROCESSING

A number of sentences throughout the text raise some concerns about the quality of the data and a possible confounding role of movement artefacts.

Lines 519-520: *"although from the raw data it was apparent that some of the subjects were periodically awake "*.

Lines 532-534: *"All the data was manually checked for larger artifacts. Especially during the N2 stage, the data from the sleeping children rarely contained any blinks, and some of the muscle and movement artifacts were attenuated by the bandpass filtering applied"*.

Lines 361-362: *"Possible differences between sexes may also be attributable to external factors, such as non-equal distribution of movement artifacts"*

Lines 433-438: *"we did not, e.g., remove noisy segments or use automated artifact rejection algorithms. Independent component analysis was applied to remove the heartbeat artefact, and bandpass filtering took out most of the slow drifts, but some muscular and ocular artefacts likely remained in the data. Any discrepancy in artefact density, such as uneven amount of movement between sexes in N1 sleep, might account for some of the observed sex differences."*

How were these artefacts and apparent periods of wakefulness distributed across subjects and ages? In particular, were the concerned subjects displaying higher accuracy (suggesting these non-brain signals contributed strongly to their "fingerprint") or lower (thus artificially reducing the decoding power of the model)? An examination of the latent components might help answering these questions.

We thank the reviewer for raising this issue. Eye-movements, blinks, and heartbeat artefacts were removed algorithmically using independent component analysis (Fast-ICA implementation on MNE Python). To see if movement/muscle artifacts measured by EMG sensors or cap size affected the results, we added these variables, together with age and sex, to the regression analysis (Pages 32-33 in Methods, lines 819-828, **Table 1** & **Figure 6, Supplementary Tables S6 & S7;** see also our answer to major revision point 9 above). When fingerprinting on N1 sleep, the muscle artifacts affected the boys' BRRR-differentiability scores more negatively than girls'. For correlation-based differentiability scores, females showed higher age effect than males. In other sleep stages, we did not observe any significant effects from these variables. We did not observe any significant positive relationships with muscle artifacts and differentiability in any of the models. Also, previous fingerprinting studies, albeit with adults or older children, have been robust against many non-brain artifacts (da Silva Castanheira, 2024). We have now updated the relevant parts in the Discussion (page 19, lines 433-438): **"We observed significant sex effects on differentiability only within N1 sleep. Sex mediated the differentiability scores slightly differently based on the used approach: for BRRR, boys' fingerprint stability was more affected by muscle artifacts than girls', whereas for correlation, the fingerprint stability of girls**

increased more rapidly than of boys as a function of age. In all sleep stages, large muscle artifacts were relatively rare across all subjects, thus making the regression fits more sensitive to outliers”.

We also tested separately if there was an age-dependency between the muscle artifact ratio and age; we found very small but significant negative trends in noise ratio as a function of age (see **Supplementary Figure S3** and below). As the effect size is vanishingly small, we do not believe that changes in the muscle artifact load explained the observed age-effect in N1 differentiability scores. However, we note that the difference in movement patterns seems to be higher in very young children, but the result might be sensitive to outliers.

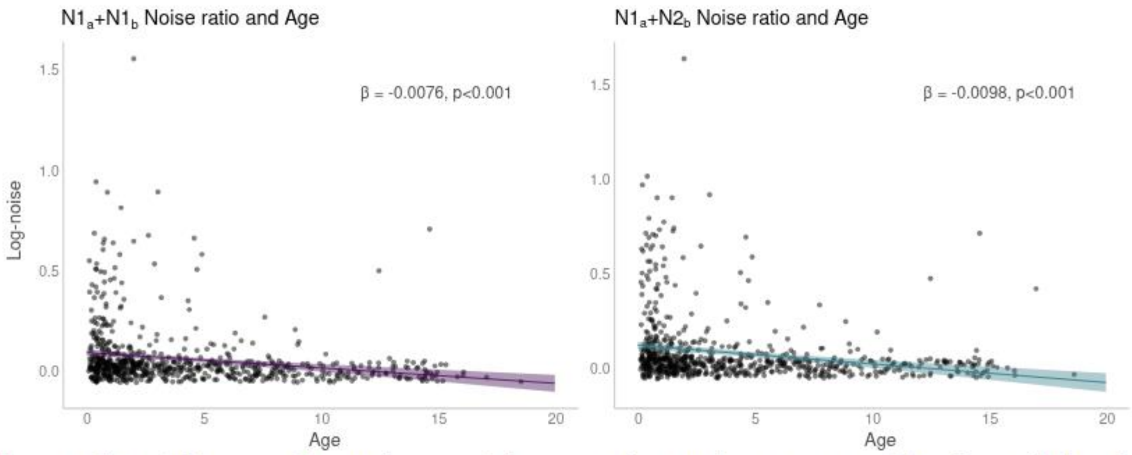

Figure S3. The age dependency of log-muscle artifact ratio in N1 sleep (left: $\beta = -0.0076, p < 0.001, r^2 = 0.045$) and mix of N1 and N2 sleep (right:$\beta = -0.0098, p < 0.001, r^2 = 0.054$).

4. CHANGES IN SPECTRAL POWER DURING CHILDHOOD

Lines 116-117 mention a pattern specific to infants <3 months. No results are displayed for this age group, and this grouping is not coherent with the age groups used for the other analyzes. Which information supports this claim?

We apologize for this mention: we have removed it from the text.

5. LANGUAGE AND WRITING

Line 19: fMRI has not been defined. Line 268: (p¿0.05)

The typos have been fixed.

References

Tapani, K. T., Nevalainen, P., Vanhatalo, S., & Stevenson, N. J. (2022). Validating an SVM-based neonatal seizure detection algorithm for generalizability, non-inferiority and clinical efficacy. Computers in Biology and Medicine, 145, 105399.

Leppäaho, E., Renvall, H., Salmela, E., Kere, J., Salmelin, R., & Kaski, S. (2019). Discovering heritable modes of MEG spectral power. Human brain mapping, 40(5), 1391-1402.

Haakana, J., Merz, S., Kaski, S., Renvall, H., & Salmelin, R. (2024). Bayesian reduced rank regression models generalizable neural fingerprints that differentiate between individuals in magnetoencephalography data. European Journal of Neuroscience, 59(9), 2320-2335.

Iyer, K. K., Roberts, J. A., Waak, M., Vogrin, S. J., Kevat, A., Chawla, J., ... & Stevenson, N. J. (2024). A growth chart of brain function from infancy to adolescence based on EEG. EBioMedicine, 102.

Pauls, K. A. M., Salmela, E., Korsun, O., Kujala, J., Salmelin, R., & Renvall, H. (2024). Human sensorimotor beta event characteristics and aperiodic signal are highly heritable. Journal of Neuroscience, 44(5).

Cooper, S. R., Jackson, J. J., Barch, D. M., & Braver, T. S. (2019). Neuroimaging of individual differences: A latent variable modeling perspective. Neuroscience & Biobehavioral Reviews, 98, 29-46.

da Silva Castanheira, J., Orozco Perez, H. D., Misic, B., & Baillet, S. (2021). Brief segments of neurophysiological activity enable individual differentiation. Nature communications, 12(1), 5713.
